# SentinelSphere: An AI-driven cybersecurity platform integrating real-time threat detection with security awareness education

**DOI:** 10.12688/openreseurope.22957.1

**Published:** 2026-02-25

**Authors:** Nikolaos D. Tantaroudas, Ilias Karachalios, Andrew J. McCracken

**Affiliations:** 1 Ethniko Metsobio Polytechneio Ereunetiko Panepistemiako Institouto Systematon Epikoinonion kai Ypologiston, Athens, Attica, Greece; 2 National Technical University of Athens, Zografou, Attica, Greece; 3 183 Rue de l'Abbé Griffon, DASKALOS-APPS, Péronnas, 01960, France

**Keywords:** Cybersecurity Awareness, Real-Time Anomaly Detection, Security Education, Large Language Models, Human-Centric Threat Intelligence, Deep Neural Networks, Traffic Light System, Cyber Resilience, Intrusion Detection Systems, SIEM

## Abstract

**Background:**

The cybersecurity domain faces dual challenges: a global shortage of qualified professionals and persistent human-factor vulnerabilities contributing to the majority of security breaches. Traditional Security Information and Event Management (SIEM) systems generate excessive false positives causing analyst alert fatigue, while conventional awareness programmes demonstrate limited effectiveness in changing user behaviour. Integrated solutions addressing both technical detection and human-centric education are needed.

**Methods:**

We developed SentinelSphere, an AI-driven platform combining machine learning-based threat detection with Large Language Model (LLM)-powered security training. The detection component employs an Enhanced Deep Neural Network trained on the CIC-IDS2017 and CIC-DDoS2019 benchmark datasets. The educational component utilises Microsoft’s Phi-4 model with quantisation techniques to enable deployment on standard hardware. System performance was optimised through Rust-based preprocessing and validated via pilot deployments with 76 stakeholders across professional and educational settings in Greece.

**Results:**

The Enhanced DNN achieved high detection accuracy with significant false positive reduction compared to baseline models, while maintaining strong recall for critical attack categories including DDoS, botnet, and brute force attacks. Rust optimisation delivered substantial speedup in both single-record and batch processing. Stakeholder validation revealed high satisfaction rates, with most participants achieving improved security concept comprehension post-training. The platform identified critical awareness gaps, particularly regarding data protection regulations and multi-factor authentication adoption.

**Conclusions:**

SentinelSphere demonstrates that integrating intelligent threat detection with adaptive, LLM-powered security education can effectively address both technical and human-factor cybersecurity challenges. The resource-efficient design enables deployment in SME environments without enterprise-grade infrastructure, supporting comprehensive cyber resilience approaches within the European Union’s regulatory framework.

## Plain Language summary

Cyberattacks are becoming more sophisticated while organisations struggle to find enough skilled cybersecurity professionals. Most existing security systems generate too many false alarms, causing analysts to miss real threats, and they do not help employees understand and recognise security risks. The system developed in this paper, addresses these problems by combining advanced threat detection with security training in a single platform. The system uses artificial intelligence to identify cyber threats with high accuracy while significantly reducing false alarms. It displays security information using a simple traffic light system, showing green for safe, yellow for caution, and red for danger, making it easy for anyone to understand the current security status. An AI-powered chatbot helps employees learn about cybersecurity through natural conversation, running efficiently on modern office computers without special hardware. The core detection system was rewritten in the Rust programming language, making it more than five times faster compared to the baseline python framework. Testing showed the system can process millions of security events quickly and accurately. Workshops with business professionals from shipping and manufacturing companies, and university students, confirmed that users found the platform easy to understand and valuable for improving their security awareness. Importantly, surveys before training revealed that most participants lacked basic security knowledge, showing exactly why tools like the one developed in this study are needed. This research demonstrates that protecting organisations from cyber threats requires not only better technology but also helping people become more security-aware.

## Introduction

### Background and motivation

The cybersecurity landscape faces an escalating crisis characterised by increasingly sophisticated attack vectors and a critical shortage of skilled security professionals. ^1^ Global estimates indicate a shortage of approximately 3.4 million cybersecurity workers, with this gap projected to grow as digital transformation accelerates across industries. ^2^ Traditional Security Information and Event Management (SIEM) systems, while effective at data aggregation, often overwhelm analysts with false positives and lack the contextual intelligence necessary for rapid threat response. ^3^ Past studies have indicated that security analysts spend up to 25% of their time investigating false positive alerts, leading to alert fatigue and increased risk of missing genuine threats. ^4^

Furthermore, the human factor remains a significant vulnerability in cybersecurity. According to the Verizon 2023 Data Breach Investigations Report, 74% of all breaches include the human element, with people being involved either via error, privilege misuse, use of stolen credentials, or social engineering. ^5^ This finding underscores a fundamental truth: technological solutions alone cannot adequately protect organisations against modern cyber threats. Effective cybersecurity requires a holistic approach that combines advanced threat detection with systematic enhancement of human security awareness.

The European Union’s approach to cybersecurity emphasises resilience in critical infrastructure protection, ^6^ while the NIST Cybersecurity Framework 2.0 provides comprehensive guidance for managing cybersecurity risks across organisations of all sizes. ^7^ The Framework’s addition of a “Govern” function in its 2024 update highlights the increasing recognition that cybersecurity governance and human factors must be integral to any security strategy. These frameworks establish the conceptual foundation upon which technical solutions must be built.

### The ResilMesh framework

The ResilMesh project, funded under the EU Horizon Europe programme (Grant Agreement No. 101119681), has established a comprehensive cybersecurity framework designed to enhance cyber resilience for dispersed, heterogeneous cyber systems. ^8^ The baseline system is built on the NATS message streaming framework, and provides secure event collection and processing capabilities while implementing resilience engineering best practices including redundancy, segmentation, and dynamic positioning. ^9, 10^

The architecture implements a sophisticated data pipeline where security events flow from collection agents through Vector (a high-performance observability data pipeline), are processed through NATS message broker for reliable, high-throughput delivery, undergo anomaly detection processing, and are stored in OpenSearch for indexing and analysis. This architecture provides the foundational infrastructure upon which SentinelSphere extends its advanced threat analytics and human-centric capabilities.

### Research contributions

This paper presents SentinelSphere, a next-generation cybersecurity platform developed as an extension to the ResilMesh framework that addresses contemporary cybersecurity challenges through an innovative dual approach: combining advanced AI-driven threat detection with integrated security awareness education. Unlike conventional solutions that treat threat detection and security training as separate domains, SentinelSphere creates a synergistic system where every security event becomes an opportunity for organisational learning and resilience building.

The primary contributions of this work are fivefold: i) **Enhanced Deep Neural Network Architecture**: An Enhanced DNN model achieves 94% F1 score while reducing false positives by 69.5% through innovative feature engineering including HTTP-specific anomaly indicators such as Request Complexity Score, Response Pattern Ratio, and Path Entropy. ii) **Traffic Light System (TLS)**: A sophisticated threat visualisation system that transforms complex threat intelligence into intuitive visual indicators (Green/Yellow/Red), democratising security understanding across technical expertise levels with validated 91.7% comprehension rates. iii) **LLM-Powered Security Education**: A Large Language Model-powered conversational agent based on Microsoft’s Phi-4, optimised through Q4_K_M quantisation (reducing model size from 28 GB to 2.5 GB) for deployment on standard enterprise hardware, providing real-time, context-aware cybersecurity guidance without requiring specialised infrastructure. iv) **Performance Optimisation**: Complete algorithm rewrite from Python to Rust achieving 5.6× average speedup for steady-state operations and up to 326× improvement for large batch processing, enabling enterprise-scale deployment. v) **Comprehensive Validation**: Multi-sector stakeholder validation through professional and educational workshops with 54 participants demonstrating 86.1% user satisfaction, 77.8% deployment interest, and identification of critical cybersecurity knowledge gaps in target populations. This work extends our preliminary findings ^11^ by incorporating detailed results from pilot demonstrations, performance optimisations through Rust implementation, and comprehensive stakeholder validation across multiple sectors.

## Related work

### Machine learning for intrusion detection

The application of machine learning to cybersecurity has evolved significantly from rule-based systems to sophisticated deep learning architectures. Early intrusion detection systems relied primarily on signature-based detection, which proved effective against known threats but failed to identify novel attack vectors. ^12^ The emergence of anomaly-based detection using machine learning addressed this limitation by learning patterns of normal behaviour and identifying deviations.

Yin et al. ^13^ demonstrated the effectiveness of Recurrent Neural Networks (RNNs) for intrusion detection, achieving 98.6% accuracy on the NSL-KDD dataset. Their work established the viability of deep learning for sequential network traffic analysis but suffered from high false positive rates in production environments due to the temporal dependencies in network traffic that RNNs capture well but also overfit to.

Vinayakumar et al. ^14^ proposed a hybrid deep learning framework combining Convolutional Neural Networks (CNNs) and Long Short-Term Memory (LSTM) networks, creating scale-hybrid-IDS-AlertNet. Their framework improved detection of zero-day attacks by capturing both spatial patterns (through CNNs) and temporal patterns (through LSTMs) in network traffic. However, the computational requirements of their approach limited practical deployment in resource-constrained environments.

The release of the CIC-IDS2017 and CIC-DDoS2019 datasets by the Canadian Institute for Cybersecurity ^15, 16^ provided the research community with realistic, labelled network traffic data for training and evaluation. These datasets contain over 400 GB of network traffic including diverse attack vectors: brute force attacks, cross-site scripting (XSS), SQL injection, distributed denial of service (DDoS), and benign traffic patterns. Sharafaldin et al. ^17^ provided detailed analysis of CIC-IDS2017, establishing benchmarks that subsequent research has sought to exceed.

Recent advances have focused on reducing false positives while maintaining detection accuracy. Khan et al. ^18^ introduced attention mechanisms for network traffic analysis, achieving 92% precision by allowing models to focus on the most relevant features of network flows. However, their approach remained limited to network-layer attacks and did not incorporate application-layer intelligence. Ferrag et al. ^19^ conducted a comprehensive survey of deep learning approaches for intrusion detection, identifying the trade-off between detection accuracy and false positive rates as a persistent challenge. Contractive autoencoders for anomaly detection have shown promise in reducing false positives. ^20^ By learning compressed representations of normal traffic patterns and detecting deviations, these approaches have achieved strong performance on the CIC-DDoS2019 dataset (97.58% accuracy) while maintaining lower false alarm rates than supervised approaches. Our work extends these approaches by incorporating application-layer features, particularly HTTP-specific patterns, to enhance detection accuracy across multiple attack vectors while significantly reducing false positive rates. The key innovation lies in the feature engineering approach that combines network-layer statistics with HTTP-specific indicators.

### Large language models in cybersecurity

Large Language Models have emerged as transformative tools across multiple domains, including cybersecurity. ^21^ Recent surveys ^22, 23^ have systematically analysed the application of LLMs to cybersecurity tasks, identifying six key domains: vulnerability detection, anomaly detection, cyber threat intelligence, penetration testing, digital forensics, and blockchain security.

The application of LLMs to cybersecurity education represents a particularly promising area. Jaffal et al. ^24^ provide a comprehensive systematic literature review covering over 300 works on LLMs in cybersecurity, identifying cybersecurity knowledge assistants as a key application area. These systems improve users’ security awareness and assist in defending against cyber-attacks through natural language interaction.

Atlam et al. ^25^ explored GPT-based systems for security questionnaire generation, demonstrating that LLMs can effectively create educational content for security awareness training. However, their deployment required significant computational resources (high-end GPUs), limiting accessibility for organisations without specialised infrastructure.

Hassanin and Moustafa ^26^ provide a comprehensive overview of LLMs for cyber defence, categorising techniques into threat intelligence, vulnerability assessment, network traffic security, privacy preservation, personnel awareness, and ethical security. Their survey highlights that LLMs can significantly improve cyber security operations and threat detection, while also noting the importance of model efficiency for practical deployment.

Chhetri ^27^ explored LLM-powered pedagogical approaches to cybersecurity education, demonstrating that LLMs can serve as effective cognitive assistants supporting interpretation, explanation, and troubleshooting in cybersecurity courses. Their framework emphasises active student engagement and verification against authoritative sources.

Our approach presented in this study, addresses the existing computational limitation of domain specific LLMs through model quantisation techniques. By applying Q4_K_M quantisation to Microsoft’s Phi-4 model, we reduce model size from 28 GB to 2.5 GB while maintaining educational effectiveness. This enables deployment on standard enterprise hardware (16 GB RAM, CPU-only), achieving 15-20 tokens per second, sufficient for interactive educational dialogue.

### Human factors in cybersecurity

The integration of human factors in cybersecurity has gained prominence following high-profile breaches attributed to social engineering. Research consistently demonstrates that technology alone cannot adequately protect organisations, and human behaviour plays a critical role in both creating vulnerabilities and implementing effective defences. ^28^

Aldawood and Skinner ^29^ conducted a comprehensive review of cybersecurity social engineering training and awareness programs, identifying common pitfalls including one-time training approaches, lack of engagement, and failure to adapt content to different user populations. Their findings emphasise the importance of continuous, interactive training that adapts to evolving threats.

Bada et al. ^30^ investigated why cybersecurity awareness campaigns fail to change behaviour, identifying key factors including information overload, lack of personal relevance, and absence of immediate feedback. Their work suggests that effective security education must be contextualised, incremental, and integrated into daily workflows, principles that inform the design of the system presented in this study.

The multimedia approach to cybersecurity awareness has been systematically reviewed by Zhang-Kennedy and Chiasson, ^31^ who found that interactive, visual approaches outperform traditional text-based training. This finding directly supports our Traffic Light System design, which transforms complex threat data into intuitive visual indicators.

### Cyber resilience frameworks

The concept of cyber resilience has evolved from traditional security approaches to encompass preparation, response, and recovery capabilities. ^32^ Unlike traditional security, which focuses primarily on prevention, resilience acknowledges that breaches are inevitable and organisations must be prepared to detect, respond to, and recover from incidents.

The cybersecurity mesh architecture concept ^33^ enables distributed security services while maintaining centralised intelligence and coordination. This architectural pattern aligns with SentinelSphere’s design as a modular extension to the ResilMesh framework, providing advanced analytics without disrupting existing security workflows.

Recent work by Somma et al. ^34^ demonstrated edge-based anomaly detection within the ResilMesh framework, highlighting the importance of distributed processing for critical infrastructure protection. Their work on smart water distribution systems showed that edge-based detection can achieve comparable accuracy to centralised approaches while reducing latency and bandwidth requirements. SentinelSphere builds upon this foundation by adding human-centric security awareness capabilities while maintaining seamless integration with the ResilMesh ecosystem.

## System architecture and design

### Overall architecture

**Figure 1.**
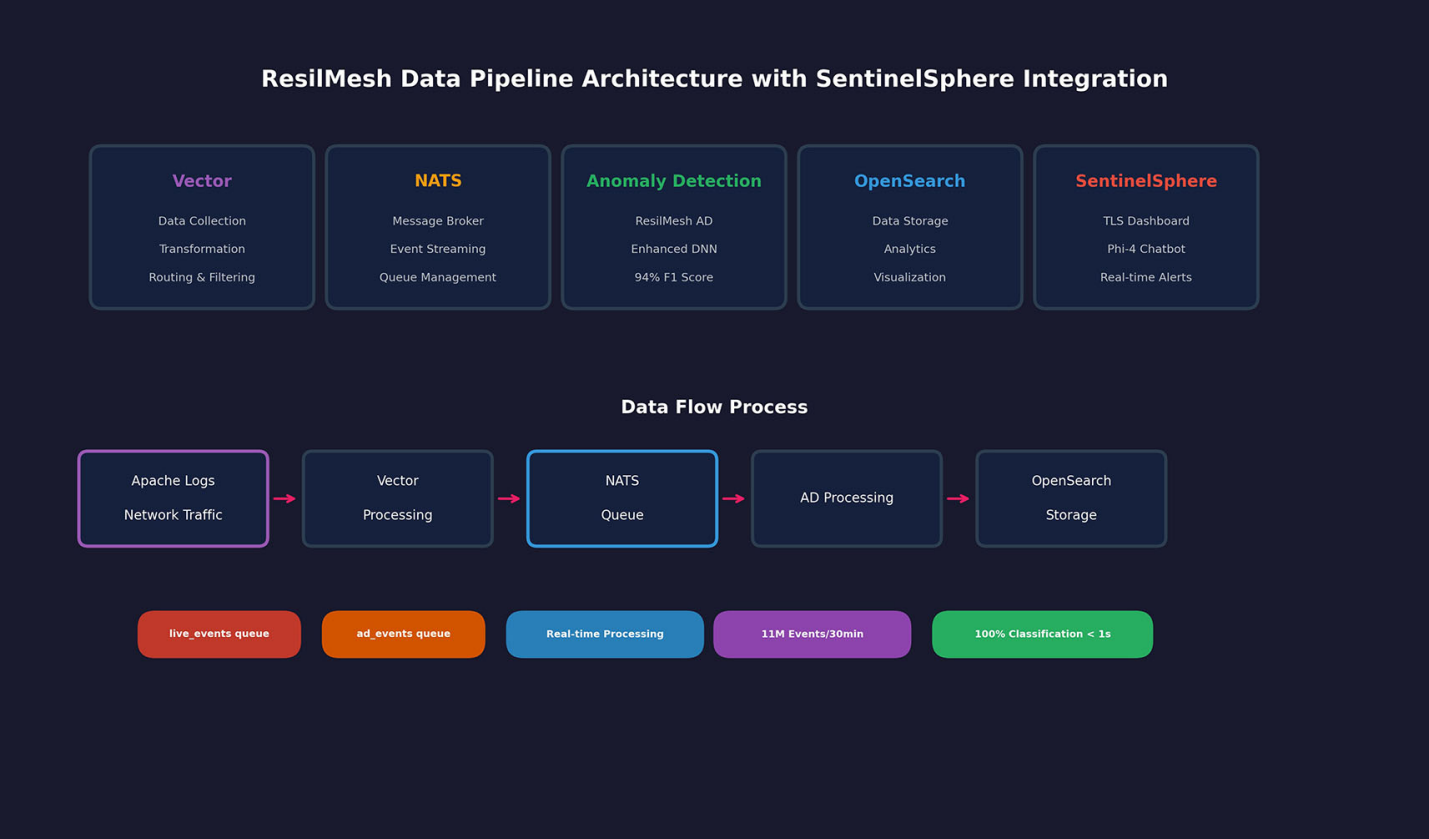
High-level system architecture showing SentinelSphere integration with ResilMesh stack.

SentinelSphere adopts a microservices architecture integrated with the ResilMesh security framework, enabling scalable, real-time processing of security events. As illustrated in Figure 1, the system comprises four primary layers: Data Ingestion, Processing and Analysis, Intelligence and Education, and Presentation.

Figure 1 illustrates the high-level system architecture, outlining where SentinelSphere integrates with the ResilMesh stack. The architecture shows security events flowing from multiple data sources through Vector for log collection, NATS message broker for reliable delivery, the Anomaly Detection module for threat identification, and finally to the SentinelSphere dashboard for visualisation and user interaction.

The architecture operates as a strategic enhancement to the ResilMesh security framework, integrating as an advanced threat analytics layer while preserving the existing architecture’s integrity. SentinelSphere operates through direct subscriptions to ResilMesh’s NATS messaging infrastructure, consuming both enriched security events (‘enriched_events’) and anomaly detection alerts (‘ad_events’). This non-intrusive approach allows SentinelSphere to function as a parallel consumer without disrupting established workflows.

**Figure 2.**
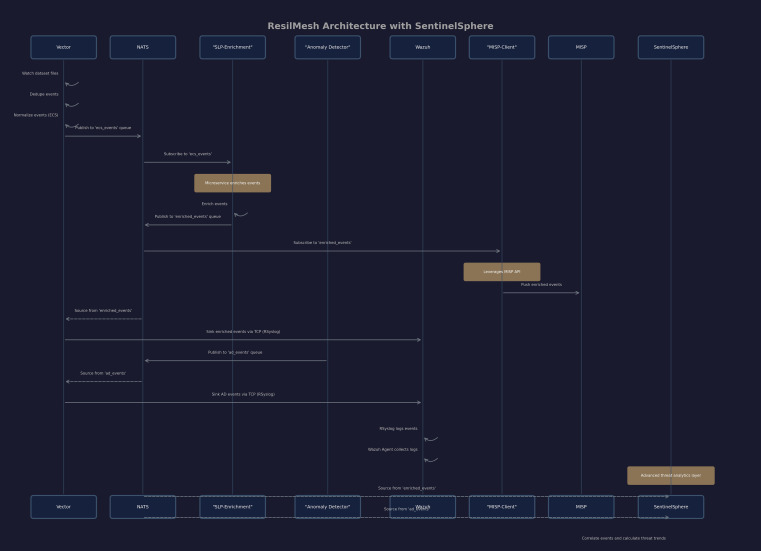
Data flow architecture - SentinelSphere and ResilMesh integration paths.

Figure 2 presents the comprehensive data flow pipeline architecture. Events move from Vector through NATS message broker to the Anomaly Detector, then through ad_events topic to the Threat Calculator, which updates Redis before displaying on the Dashboard. The diagram illustrates the integration paths between SentinelSphere and ResilMesh components.

The Data Ingestion layer utilises Vector for log collection and transformation, processing diverse data sources including network traffic, application logs, and system events. Vector serves as the primary data collection and transformation layer, ingesting security events from multiple sources and performing real-time transformations including routing, logging, fusion, filtering, augmentation, reduction, and monitoring. Events flow through the NATS message broker, ensuring reliable, high-throughput message delivery to downstream components. This architecture supports processing rates exceeding 5,000 events per second on standard hardware.

### Enhanced deep neural network model

The core of SentinelSphere’s threat detection capability is an Enhanced Deep Neural Network model that extends traditional network traffic analysis with application-layer intelligence. The model architecture consists of a 64-64-output neural network enhanced with 31 features: 21 standard network features plus 10 HTTP-specific indicators.

The standard network features include flow duration, total forward and backward packets, packet length statistics (mean, maximum, minimum, standard deviation), flow bytes per second, flow packets per second, inter-arrival time statistics, flag counts (SYN, FIN, RST, PSH, ACK, URG), and active/idle time statistics.

The HTTP feature engineering includes: i) Request Complexity Score: Quantifies HTTP request sophistication by analysing URL length, parameter count, header complexity, and payload characteristics. Advanced attacks often employ complex requests to exploit parsing vulnerabilities or evade simple pattern matching. Scores range from 0 (simple) to 1 (highly complex). ii) Response Pattern Ratio: Analyses server response patterns to detect successful exploits by comparing response sizes, status codes, and timing patterns against baseline normal behaviour. Abnormal ratios may indicate successful exploitation or data exfiltration. iii) Path Entropy: Estimates randomness in request paths using Shannon entropy, detecting obfuscation attempts common in automated attacks. Normal human browsing typically exhibits lower entropy than automated scanning or fuzzing tools. iv) Automated Tool Detection: Binary indicator identifying requests from automated security tools, scanners, or bots based on User-Agent strings, request timing patterns, and request structure characteristics.

Attack-Specific Pattern Recognition: Binary indicators for SQL injection (detecting common injection patterns like UNION SELECT, OR 1=1), cross-site scripting (XSS) (detecting script tags, event handlers, and encoded payloads), and brute force patterns (detecting high-frequency authentication attempts from single sources).

For the training of the Neural Network we utilised the CIC-IDS2017 and CIC-DDoS2019 datasets, comprising approximately 400 GB of labelled attack data including: i) Web Attack-Brute Force: 1,507 samples; ii) Web Attack-XSS: 652 samples; iii) Web Attack-SQL Injection: 21 samples; iv) Benign Traffic: 168,186 samples.

Subsequently, the model hyperparameters were optimised through extensive experimentation using grid search: i) Batch size: 32 for efficient GPU utilization; ii) Learning rate: 0.001 with Adam optimiser for stable convergence; iii) Dropout rate: 0.2 preventing overfitting; iv) L2 regularisation: 0.001 for weight constraint; v) Maximum epochs: 50 with early stopping (patience=5); vi) Decision threshold: 0.4 balancing precision and recall.

### Traffic light system

**Figure 3.**
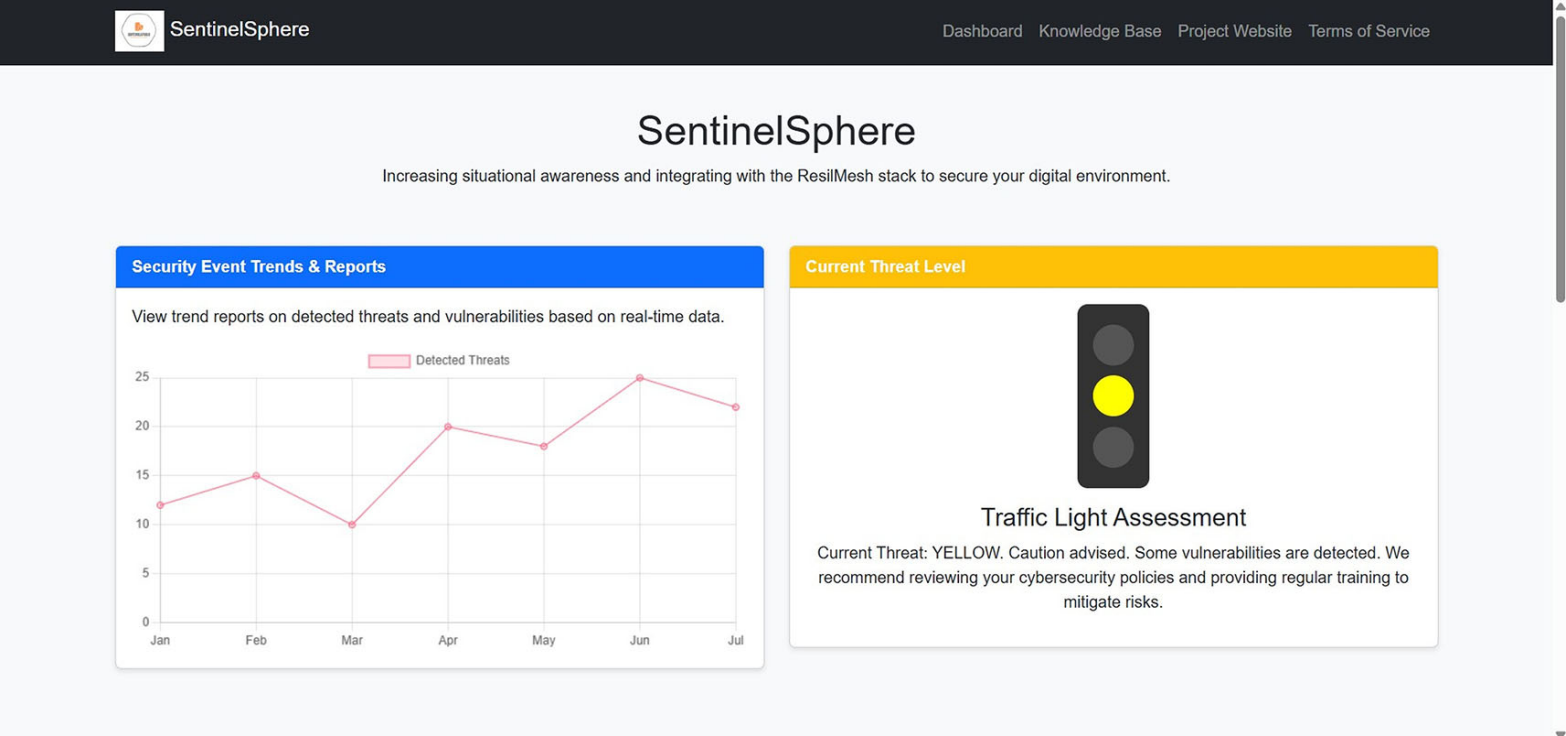
SentinelSphere dashboard showing traffic light assessment and event trends & reports chart.

The Traffic Light System provides intuitive threat visualisation through a sophisticated scoring algorithm that transforms complex security telemetry into actionable indicators accessible to users across all technical expertise levels. Figure 3 displays the SentinelSphere Dashboard interface featuring the Traffic Light Assessment panel on the right side showing the current threat level status (Green/Yellow/Red), and the Security Event Trends & Reports chart on the left displaying detected threats over time. The interface provides at-a-glance security posture assessment alongside detailed trend analysis.

The system processes anomaly detection events and calculates threat scores from 0-100, determining dashboard status: i) **GREEN (0-30%)**: Normal operation with low threat activity; ii) **YELLOW (30-70%)**: Elevated threat level requiring attention; iii) **RED (70-100%)**: Critical security events demanding immediate response

**Figure 4.**
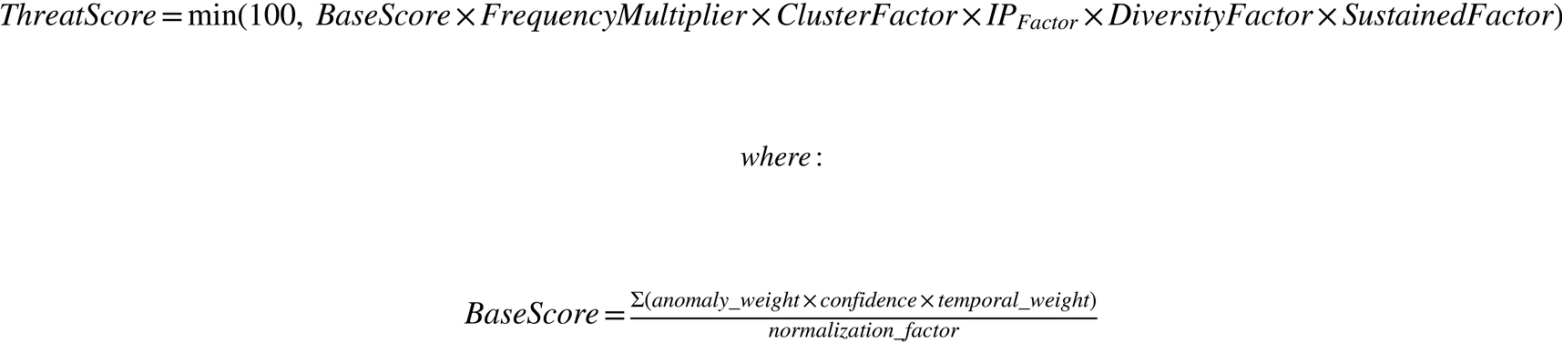
Traffic light threat calculation equation showing the mathematical formula.

Figure 4 presents the mathematical formula for calculating the final threat score. The equation Final_Score = min(100, Base_Score × Frequency_Multiplier × Cluster_Factor × IP_Factor × Diversity_Factor × Sustained_Factor) incorporates multiple factors to produce a comprehensive threat assessment. Table 1 presents the complete parameter definitions for the Traffic Light System scoring algorithm, including the values and justifications for each parameter.

**Table 1.** Parameters definition of traffic light system.

Parameter	Description	Value range
BaseScore	Weight sum of detected anomalies normalized to 0-100 scale	0-100
anomaly_weight	Severity weight per threat type (e.g., 0.95 for data exfiltration, 0.90 for unauthorized access, 0.85 for DoS)	0.3-0.95
confidence	Detection confidence score	0.5-1.0
temporal_weight	Time-based decay factor for older events	0-1.0
Normalization_factor	Scaling factor to maintain 0-100 range	Calculated
FrequencyMultiplier	Event rate per minute (1.0x for 1-5 events, 1.5x for 5-20, 2.0x for 20-50, 3.0x for >50)	1.0-3.0x
ClusterFactor	Temporal clustering (1.2x for single cluster, 1.5x for 2 clusters, 2.0x for 3+ clusters)	1.0-2.0x
IPFactor	Attack source concentration (1.5x single IP, 1.3x for 2-4 IPs, 1.0x distributed)	1.0-1.5x
DiversityFactor	Number of different attack types (1.8x for 5+ types, 1.4x for 3-4, 1.2x for 2, 1.0x for 1)	1.0-1.8x
SustainedFactor	Duration of continuous attack activity (2.0x for >3 min high-rate, 1.5x for >2 min, 1.2x for >1 min)	1.0-2.0x

### LLM-powered cyber-security education

**Figure 5.**
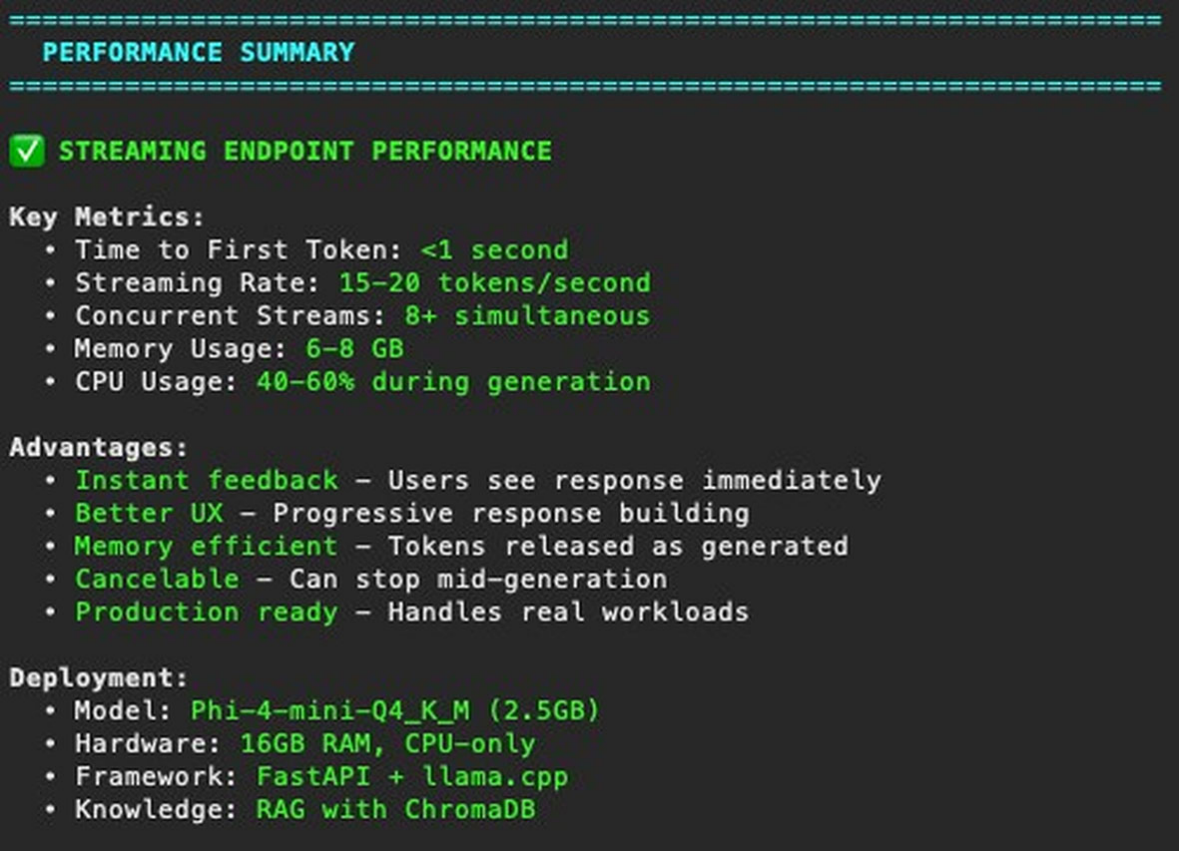
Cybersecurity conversation agent - Phi-4 model performance summary.

SentinelSphere incorporates Microsoft’s Phi-4 language model, optimised for cybersecurity contexts through domain-specific fine-tuning and deployment optimisation. The model underwent Q4_K_M quantisation, reducing size from 28 GB to 2.5 GB while maintaining educational effectiveness. Figure 5 shows the performance summary for the Phi-4 cybersecurity conversation agent, displaying streaming endpoint performance metrics: time to first token <1 second, streaming rate 15-20 tokens/second, concurrent streams 8+ simultaneous, memory usage 6-8 GB, and CPU usage 40-60% during generation. The deployment specifications show Model: Phi-4-mini-Q4_K_M (2.5 GB), Hardware: 16 GB RAM, CPU-only, Framework: FastAPI + llama.cpp, Knowledge: RAG with ChromaDB.

**Figure 6.**
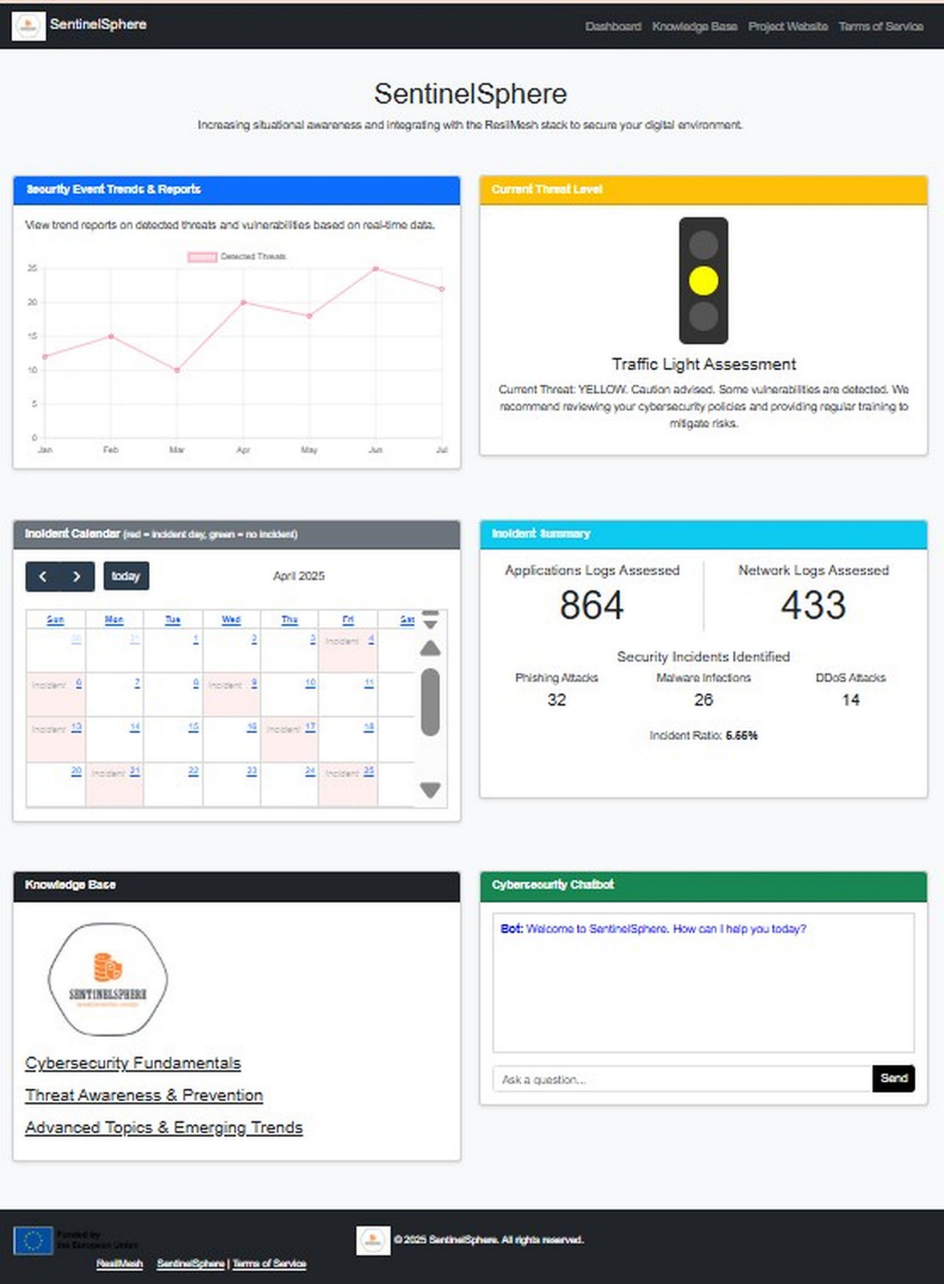
SentinelSphere dashboard-HomePage and chatbot interaction panel.

Figure 6 presents the complete SentinelSphere Dashboard homepage featuring the integrated chatbot interaction panel in the bottom-right corner. The interface displays the incident calendar for temporal analysis, security incident summary statistics showing Applications Logs Assessed and Network Logs Assessed, Security Incidents Identified (Phishing Attacks, Malware Infections, DDoS Attacks), and the Knowledge Base section providing access to cybersecurity fundamentals, threat awareness & prevention, and advanced topics & emerging trends.

**Figure 7.**
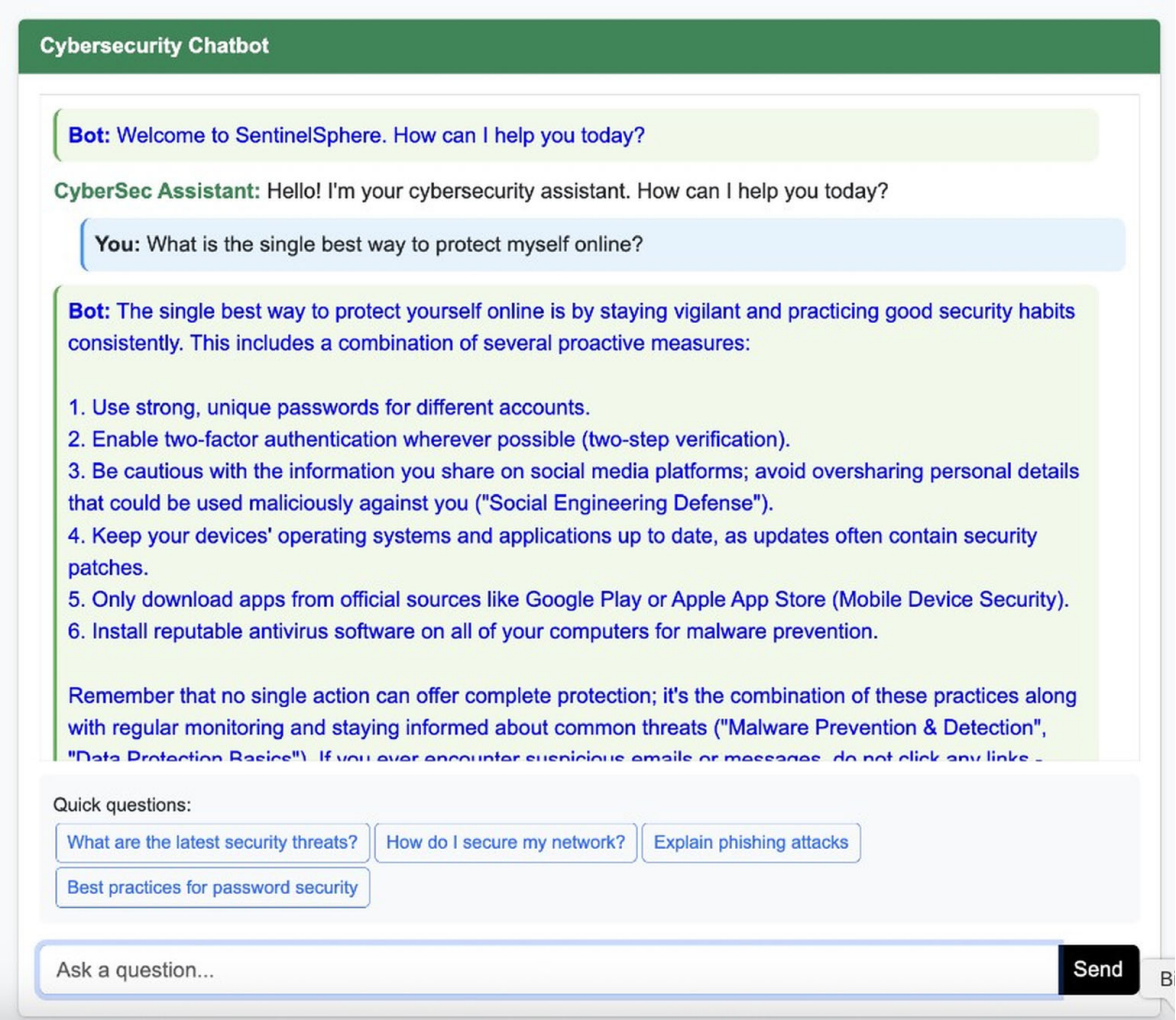
Demonstration of Phi-4 cybersecurity domain-specific LLM response.

Figure 7 demonstrates the Phi-4 cybersecurity chatbot providing comprehensive guidance on staying safe from cyber threats. The chatbot provides comprehensive cybersecurity guidance, explaining best practices for online protection including strong passwords, two-factor authentication, cautious information sharing, system updates, app downloads from official sources, and security software installation. The chatbot successfully handles complex cybersecurity queries while maintaining conversational flow and providing actionable recommendations tailored to user expertise levels.

Key implementation features include: i) **Streaming endpoint**: Time to first token <1 second for responsive interaction; ii) **Throughput**: 15-20 tokens/second on CPU, sufficient for natural conversation; iii) **Concurrency**: 8+ simultaneous streams supported; iv) **Memory footprint**: 6-8 GB during active generation; v) **Knowledge base**: Retrieval Augmented Generation (RAG) with ChromaDB for domain-specific accuracy; The implementation has been deployed to Hugging Face Hub (daskalos-apps/phi4-cybersec-Q4_K_M) for automated download and version control, with the complete implementation available in a public GitHub repository ( https://github.com/Daskalos-Apps/cybersecurity-chatbot ).

### Dashboard implementation

**Figure 8.**
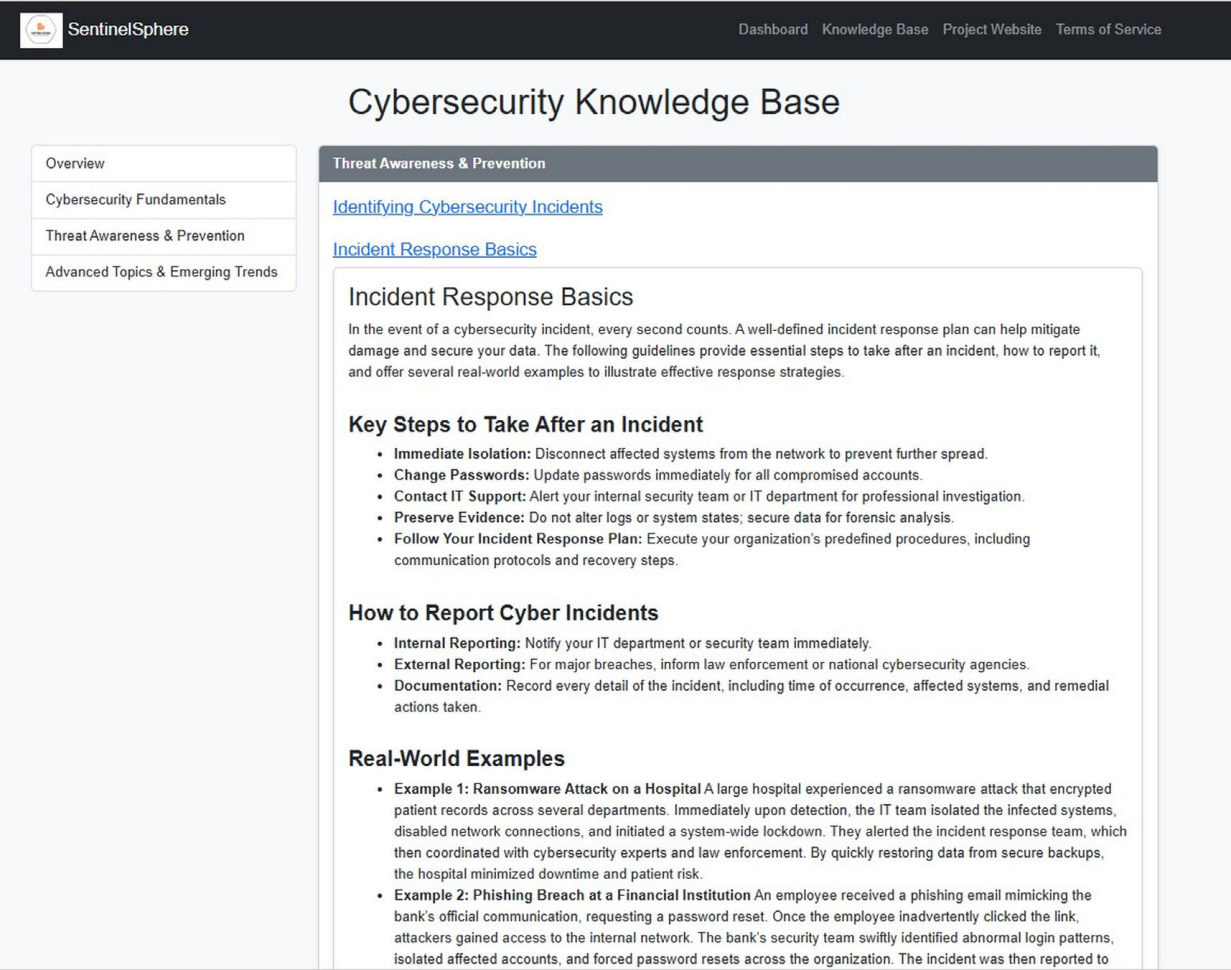
SentinelSphere dashboard cybersecurity knowledge base section.

The SentinelSphere dashboard provides comprehensive security visualisation and interaction capabilities through a web-based interface built with FastAPI and vanilla JavaScript using Jinja templating. Figure 8 shows the Knowledge Base section of the SentinelSphere dashboard, providing structured access to educational cybersecurity content organised into three main categories: Cybersecurity Fundamentals, Threat Awareness & Prevention, and Advanced Topics & Emerging Trends. This section enables users to proactively improve their cybersecurity awareness. The dashboard implements key functionalities including: i) **Real-time event viewing**: Instant visibility of incoming security incidents; ii) **TLS indicator panel**: Dynamic threat level display (Green/Yellow/Red); iii) **Forecast and trend charts**: Visual depiction of security trends and incident forecasts; iv) **Interactive chatbot panel**: Real-time AI assistance and user query handling; v) **Incident calendar**: Temporal analysis with colour-coded incident markers; vi) **Knowledge base**: Structured educational content for proactive learning.

## Implementation and performance optimisation

### Rust-based performance optimisation

The core anomaly detection algorithm, initially implemented in Python during early development phases and presented in, ^11^ was completely rewritten in Rust to achieve enterprise-scale performance requirements. This strategic decision was driven by several factors: i) Enhanced performance for enterprise-scale event processing; ii) Reduced computational resource requirements; iii) Improved memory safety and concurrency handling; iv) Better integration with high-throughput data pipelines.

The rewrite process maintained algorithmic equivalence with the Python implementation while leveraging Rust’s performance characteristics through: i) Direct translation of core detection logic; ii) Optimisation of memory management through Rust’s ownership system; iii) Parallel processing implementation using Rust’s concurrency primitives; iv) Zero-cost abstractions for maintainable high-performance code.

Performance testing validated 100% accuracy equivalence between implementations while demonstrating dramatic performance improvements. The benchmarking methodology employed two distinct test configurations: i) **Steady-state performance testing**: 50 iterations per event volume (100-2,000 events) measuring typical operational behaviour; ii) **Large batch processing**: 2 iterations per volume (1,000-100,000 events) evaluating scalability under enterprise workloads.

The Rust implementation achieved a 5.6× speedup for steady-state workloads (typical operational scenarios) and dramatic speedups ranging from 4.3× to 326× for large batch processing, while maintaining 100% algorithmic accuracy with no degradation from the Python version. Table 2 presents the detailed performance comparison between Python and Rust implementations across different event volumes.

**Table 2.** Performance comparison between python and rust implementations.

Test configuration	Events	Python (ms)	Rust (ms)	Speedup
Steady-State Performance (50 iterations)	100	2.24 ms	0.59	3.8x
	500	44.13 ms	10.48	4.2x
	1000	157.07 ms	27.69	5.7x
	2000	610.01 ms	69.86	8.7x
	Average	5.6x		
Large Batch Processing (2 iterations)	1,000	0.37 sec	0.09 sec	4.3x
	5,000	7.34 sec	0,59 sec	12.5x
	10,000	25.77 sec	0.94 sec	27.5x
	50,000	558.98 sec	3.68 sec	151.9x
	100,000	1756.9 sec	5.39 sec	326.0x

Technical analysis reveals the performance gains derive from: i) Compilation to native machine code eliminating interpreter overhead; ii) Deterministic memory allocation without garbage collection pauses; iii) Efficient data structures with contiguous memory layout (VecDeque vs Python’s deque); iv) SIMD vectorisation for numeric operations; v) Function inlining that reduces per-event processing from approximately 314,000 CPU cycles (Python) to 6,375 CPU cycles (Rust) at steady-state.

### System resource utilisation

Docker container performance metrics revealed exceptional resource efficiency across all components. The Dashboard container consumed only 15% CPU and 512MB memory with a 3-second startup time. The Phi-4 Chatbot, despite its sophisticated AI capabilities, required just 40% CPU and 2.5 GB memory with an 8-second initialisation period. Infrastructure components demonstrated even greater efficiency, with Vector using 20% CPU and 256MB memory starting in 2 seconds, NATS requiring only 10% CPU and 128MB memory with 1-second startup, and OpenSearch consuming 25% CPU and 2 GB memory while initialising in 10 seconds. Table 3 shows the system resource utilisation.

**Table 3.** System resource utilisation.

Component	CPU usage	Memory	Startup time
Dashboard	15%	512 MB	3 sec
Phi-4 Chatbot	40%	2.5 GB	8 sec
Vector	20%	256 MB	2 sec
NATS	10%	128 MB	1 sec
OpenSearch	25%	2 GB	10 sec

**Figure 9.**
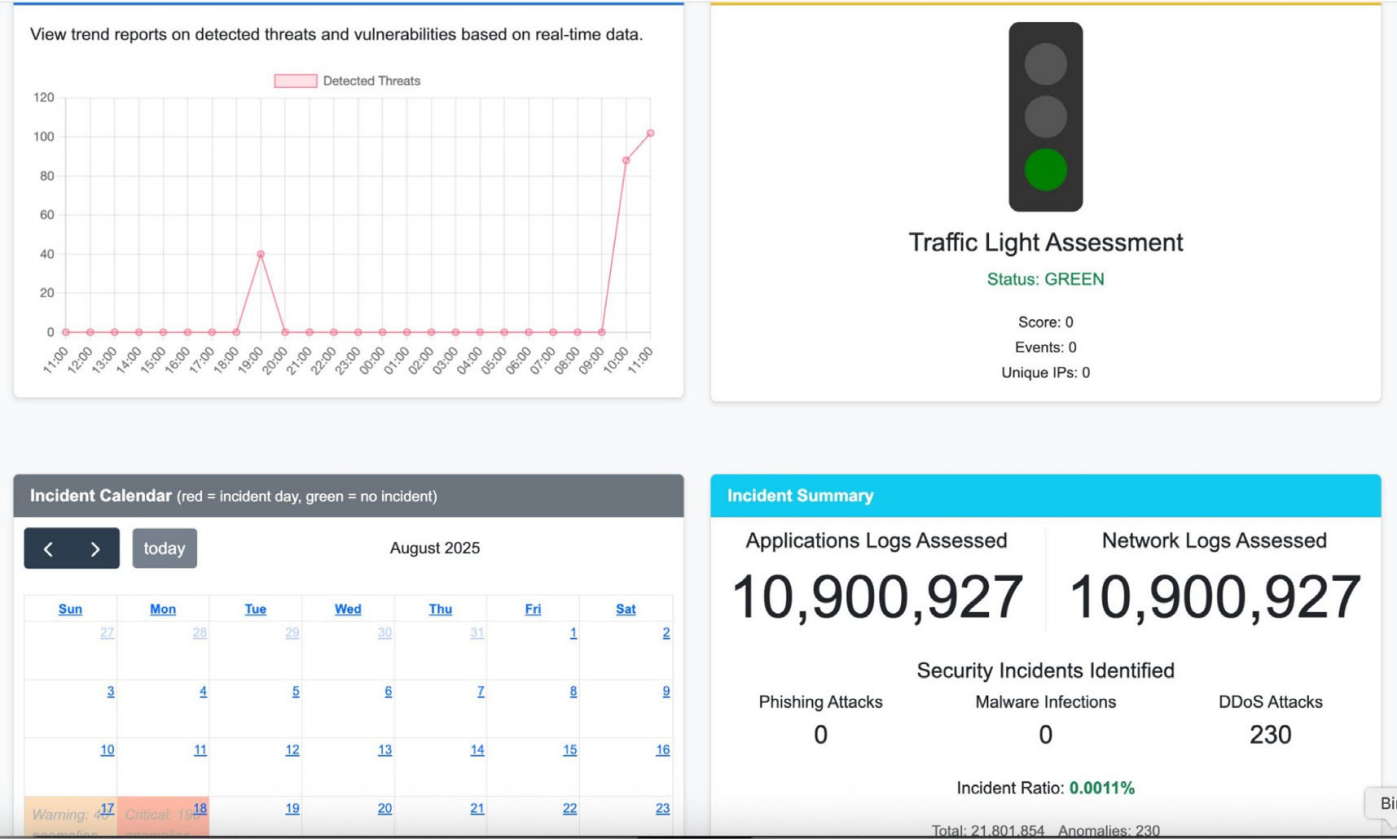
SentinelSphere scalability-processing almost 11 million of requests in approximately 30 minutes.

Real-time processing metrics further confirm the operational readiness of the system: i) Classification speed: 100% of events processed within 1 second (exceeding 95% target); ii) Event throughput: >5,000 events per second sustained (5× the 1,000 events/second requirement); iii) Pipeline latency: <100 milliseconds throughout testing; iv) False positive rate: 0.7% (significantly below 5% acceptable limit); v) Memory usage: <8 GB RAM even under peak load; vi) CPU utilisation: 60% average on 4-core system (substantial headroom); vii) Network bandwidth: 100 Mbps throughput sustained;

Figure 9 shows the scalability testing results displaying the SentinelSphere dashboard during load testing. The Incident Summary panel shows 10,900,927 Applications Logs Assessed and 10,900,927 Network Logs Assessed, with Security Incidents Identified showing 0 Phishing Attacks, 0 Malware Infections, and 230 DDoS Attacks, with an Incident Ratio of 0.0011%. The Traffic Light Assessment displays GREEN status with Score: 0, Events: 0, Unique IPs: 0, demonstrating successful processing of nearly 11 million events.

## Experimental evaluation

### Threat detection performance

The Enhanced DNN-AD model demonstrates significant improvements over baseline approaches. Table 4 presents comprehensive performance metrics comparing the enhanced model against the original ResilMesh anomaly detection baseline.

**Table 4.** Enhanced DNN model performance metrics.

Performance metric	Baseline model	Enhanced DNN	Improvement
F1 Score	91.1%	94%	3.3%
Accuracy	96.4%	97.7%	1.3%
Precision	91%	96.9%	6.5%
Recall	91.1%	91.3%	0.2%
False Positive Rates	2.3%	0.7%	69.5%
False Negative	8.9%	8.7%	1.7%
False Positives	59	19	67.8%
False Negatives	58	57	1.7%
Detected Brute Force attacks (1507 samples)	90.3%	91.2%	0.9%
Detected XSS attacks (652 samples)	91.8%	94.4%	2.6%
Detected SQL injection (21 samples)	85.7%	90.5%	4.8%

Most significantly, the enhanced model demonstrates a 69.5% reduction in false positives (from 59 to 19), while maintaining a low false negative rate. The confusion matrices reveal that the enhanced model correctly identifies 96.9% of flagged threats compared to 91.0% for the baseline model, making it production-ready with minimal alert fatigue for Security Operations Centre (SOC) teams. The HTTP-specific feature engineering provides the greatest contribution to false positive reduction. By understanding the semantics of HTTP requests rather than just statistical patterns, the model can distinguish between legitimate complex requests, such as search queries with multiple parameters, and actual attack attempts.

### Scalability testing

The platform successfully processed nearly 11 million events (10,900,927) in approximately 30 minutes, representing three months of Apache logs from production environments. This demonstrates sustained processing exceeding 5,000 events per second on standard desktop hardware. Table 5 below provides the scalability benchmarks achieved compared to the desired targets.

**Table 5.** Scalability benchmarks.

Metric	Target	Achieved
Events Processed	10 million	10,900,927
Processing Time	30 minutes	~30 minutes
Throughput	1,000 events/sec	>5,000 events/sec
Classification Latency	<1 second (95%)	<1 second (100%)
Pipeline Latency	<500 ms	<100 ms
Concurrent Connections	1,000	1,000+

System scalability benchmarks confirmed enterprise readiness through multiple stress tests. The platform successfully handled over 1,000 simultaneous connections without degradation, demonstrating deployment feasibility on standard hardware with substantial headroom for traffic spikes.

## Stakeholder validation

### Professional workshop

A targeted professional workshop on 14 October 2025 provided SentinelSphere’s first validation in real-world business contexts with three professionals from maritime, educational, and manufacturing sectors. The session included live platform demonstration with realistic threat scenarios, interactive exploration focusing on sector-specific security challenges, discussion of deployment requirements and integration capabilities, and exploration of customisation opportunities for different industry needs.

Key qualitative outcomes included validation of platform applicability across diverse sectors, identification of sector-specific customisation requirements, establishment of initial partnership discussions, and confirmation that the Traffic Light System’s simplicity resonates with non-technical decision-makers.

### Educational workshop

In collaboration with the Pedagogy Department at the University of Western Macedonia on 31 October 2025, SentinelSphere conducted an educational workshop focusing on practical cybersecurity training. A total of 76 participants attended, including pedagogy students and faculty members. The workshop followed a structured three-phase approach.

1. **Phase 1: Pre-workshop Assessment**: i) GDPR-compliant consent procedures, a baseline anonymous questionnaire assessed initial cybersecurity awareness; ii) 39 students completed the detailed pre-workshop survey; iii) Assessment covered: technology experience, AI familiarity, phishing awareness, and GDPR knowledge.
2. **Phase 2: Platform Demonstration and Training**: i) Extended presentation covering cybersecurity fundamentals and threat landscape; ii) live demonstration of the Traffic Light System and real-time threat detection; iii) interactive chatbot sessions where students tested the LLM-powered cybersecurity assistant; iv) Hands-on exploration of platform features with guided scenarios.
3. **Phase 3: Post-Workshop Evaluation:** i) distribution of detailed anonymous feedback questionnaire; ii) 36 students, completed post-workshop evaluation; iii) Assessment covered: presentation clarity, chatbot usability, feature understanding, and deployment interest.

Table 6 below provides the workshop results summary.

**Table 6.** University of Western Macedonia educational workshop results summary.

Category	Metric	Responses	N
PRE-WORKSHOP BASELINE ASSESSMENT			
Demographics	Age Distribution 18-20 years	94.9%	39
	Age Distribution (27+ years)	5.1%	39
Technology Competency	Basic Technology Use	53.8%	39
	Moderate Technology Experience	33.3%	39
	Advanced Technology Experience	10.3%	39
	No Technology Experience	10.3%	39
AI Familiarity	Frequent AI Tool Usage	51.3%	39
	Occasional AI Tool Usage	43.6%	39
	Never Used AI Tools	5.1%	39
Cybersecurity Awareness	Recognised Phishing Emails	23.1%	39
	Uncertain about Phishing	25.6%	39
	No Phishing Experience	51.3%	39
Security Practices	Password Reuse Across Sites	59%	39
	2FA Not Enabled/Unknown	84.6%	39
	2FA Enabled on All Accounts	7.7%	39
Professional Relevance	Digital Security “Very Important” for Teachers	87.2%	39
	Digital Security ‘Moderately Important’	12.8%	39
Regulatory Knowledge	No GDPR Knowledge	92.3%	39
	Heard of GDPR but Lack Details	7.7%	39
POST-WORKSHOP PLATFORM EVALUATION			
Presentation Quality	Average Comprehensibility Score	8.72/10 (87.2%)	36
	Median Score	9/10	36
	Scores 8-10 (High Comprehensibility)	91.7%	36
Platform Engagement	Actively Used SentinelSphere Chatbot	91.7%	36
	Did not Use Chatbot	8.3%	36
Chatbot Performance	Ease of Use (Mean Score)	4.06/5 (81.2%)	35
	Usefulness & Clarity (Mean Score)	3.82/5 (76.4%)	35
	Would Use Again (Mean Score)	3.82/5 (76.4%)	35
Traffic Light System	‘Very Clear’ Understanding	41.7%	36
	‘Clear’ Understanding	41.7%	36
	Total Clear/Very Clear	91.7%	36
	‘Moderate’ Understanding	8.3%	36
Deployment Interest	Definitely ‘Yes’ as Educator	16.7%	36
	‘Probably Yes’ as Educator	61.1%	36
	‘Don’t know’	13.9%	36
	‘Probably No’ or ‘Definitely No’	8.3%	36
Overall Satisfaction	Mean Satisfaction Score	4.3/5 (86.1%)	36
	Scores 4-5 (High Satisfaction)	72.2%	36
	Score 3 (Neutral/Good)	27.8%	36
Recommendation Intent	Mean Recommendation Score	3.82/5 (76.4%)	36
	Scores 4-5 (Would Recommend)	61.1%	36
	Score 3 (Neutral)	30.6%	36
	Scores 1-2 (Would not Recommend)	8.3%	36

The workshop findings were the following: i) High Engagement: 91.7% of participants actively used the chatbot during the workshop, indicating strong interest in AI-assisted cybersecurity learning; ii) Strong Comprehension: 91.7% found the Traffic Light System clear or very clear, validating the effectiveness of the visual threat communication approach for non-technical audiences; iii) Deployment Interest: 77.8% expressed interest in using the platform as educators (16.7% “Definitely Yes” + 61.1% “Probably Yes”), suggesting significant potential for cascading cybersecurity awareness through educational institutions; iv) High Satisfaction: 86.1% overall satisfaction with 72.2% rating the platform 4-5 out of 5, demonstrating strong user acceptance.

In addition, the pre-workshop assessment revealed the following critical knowledge gaps among participants: i) 92.3% had no GDPR knowledge (despite being EU citizens subject to GDPR); ii) 84.6% did not use two-factor authentication; iii) 59.0% reused passwords across sites; iv) Only 23.1% could recognise phishing emails; v) 51.3% had never encountered or couldn’t identify phishing attempts.

These findings validate the critical need for accessible cybersecurity education tools like SentinelSphere, particularly for populations who will influence future generations (educators).

## Discussion

### Addressing the human factor

SentinelSphere’s integration of threat detection with security education represents a novel use case in cybersecurity defence. Traditional approaches treat security awareness as separate from operational security, creating disconnects between threat detection and human response. Our unified approach ensures every security event contributes to organisational learning, progressively building cyber resilience. The workshop results are particularly significant in this content. The pre-workshop assessment revealed that 92.3% of future educators had no GDPR knowledge and 84.6% did not enable two-factor authentication, statistics that highlight the critical need for accessible cybersecurity education tools. These individuals will be responsible for educating the next generation, making their cybersecurity literacy essential for societal resilience.

The LLM-powered chatbot democratises security expertise, enabling non-technical users to understand and respond to threats effectively. By operating on standard hardware with only 16 GB RAM and CPU-only requirements, the solution remains accessible to organisations without specialised infrastructure, addressing the cybersecurity skills gap affecting many enterprises. Furthermore, the reported 81.2% ease-of-use rating and 76.4% usefulness rating from the workshop participate validate this approach. Post-workshop, 77.8% of participants expressed interest in deploying the platform in educational contexts. This finding suggests potential for multiplicative impact: by equipping educators with cybersecurity knowledge and tools, SentinelSphere can indirectly reach thousands of students through each trained teacher.

### Practical deployment considerations

The 69.5% reduction in false positives has profound implications for SOC operations. Security analysts spend significant time investigating false alerts, leading to alert fatigue and missed genuine threats. Past studies have indicated that analysts may review over 10,000 alerts daily, with false positive rates sometimes exceeding 50%. ^35^ SentinelSphere’s enhanced precision enables analysts to focus on legitimate threats, improving both efficiency and effectiveness.

The Traffic Light System’s intuitive visualisation facilitates rapid threat assessment across organisational hierarchies. The 91.7% comprehension rate among workshop participants, predominantly non-technical pedagogy students, validates the system’s effectiveness in democratising security understanding. Executive stakeholders can understand security posture without technical expertise, enabling informed decision-making during critical incidents.

The performance optimisation through Rust implementation addresses the key practical concern relating to the system’s potential for enterprise scalability. The 5.6× average speedup for steady-state operations ensures the system can handle typical organisational workloads, while the 326× improvement for large batch processing enables rapid analysis of historical data during incident investigations or compliance audits.

### Comparison with related work

The system presented in this study has a better performance compared to related approaches in the literature. The detection performance achieved of 94% F1 score exceeds the precision achieved with attention mechanisms in earlier studies, ^18^ while the current 69.5% false positive reduction significantly outperforms typical DNN-based approaches that achieve 5-15% reduction. ^19^ Furthermore, past studies have used GPT-based system requiring high-end GPUs, ^25^ whereas our quantised Phi-4 deployment runs on standard hardware while maintaining educational effectiveness, as validated by 76.4% usefulness ratings. Finally, the 86.1% satisfaction rate and 91.7% traffic-light-system (TLS) comprehension rate exceed typical cybersecurity tool adoption metrics, which often struggle with user acceptance due to complexity. ^30^

### Limitations and future work

While SentinelSphere demonstrates significant advances, several areas warrant future investigation: i) **Protocol Coverage**: The current implementation focuses on HTTP-based attacks. Extension to other protocols (DNS tunnelling, SMTP-based threats, industrial control system protocols like Modbus and OPC-UA) would enhance coverage for critical infrastructure environments. ii) **Knowledge Base Currency**: The chatbot’s knowledge base, while comprehensive for common threats, requires continuous updating for emerging attack vectors such as AI-generated phishing, prompt injection attacks, and novel malware variants. iii) **Longitudinal Studies**: Workshop validation captured immediate reactions; longitudinal studies tracking behavioural change over time would provide stronger evidence for educational effectiveness. iv) **Federated Learning**: Future work will explore federated learning approaches, enabling organisations to benefit from collective threat intelligence while maintaining data privacy. This is particularly relevant for sectors with sensitive data (healthcare, finance) that cannot share raw security telemetry. v) **Automated Response Integration**: Integration with automated response systems (SOAR platforms) could further reduce response times for known attack patterns, creating a complete detect-respond-educate cycle.

## Conclusions

This paper presented SentinelSphere, an innovative cybersecurity platform that successfully bridges the gap between advanced threat detection and human security awareness. Through integration with the ResilMesh framework and implementation of Enhanced Deep Neural Networks, intuitive threat visualisation, and AI-powered security education, the system addresses critical challenges in modern cybersecurity defence. The key achievements presented in this paper include: **i) 94% F1 score** in threat detection with **69.5% reduction in false positives**, enabling SOC teams to focus on genuine threats **5.6× average performance improvement** through Rust optimisation, with up to 326× speedup for batch processing; ii) Successful processing of **nearly 11 million events in 30 minutes**, demonstrating enterprise scalability; iii) Deployment of sophisticated AI capabilities on **standard enterprise hardware** (16 GB RAM, CPU-only); iv) Validation through professional and educational workshops with **86.1% user satisfaction** and **91.7% Traffic Light System comprehension;** v) Identification of critical cybersecurity knowledge gaps (92.3% lacking GDPR knowledge, 84.6% not using 2FA) validating the need for integrated education tools; vi) **77.8% deployment interest** among educators, suggesting potential for multiplicative societal impact.

SentinelSphere’s dual approach, treating every security event as both a threat to mitigate and an opportunity to educate, represents a novel solution in cybersecurity philosophy. By democratising security understanding and making advanced threat intelligence accessible across expertise levels, the platform contributes to building more resilient organisations capable of defending against evolving cyber threats.

In addition, the successful integration with ResilMesh validates the importance of modular, extensible cybersecurity architectures that can accommodate innovative solutions while maintaining operational stability. As cyber threats continue to evolve, platforms like SentinelSphere that combine technological advancement with human empowerment will be essential for maintaining effective cyber defence.

This work builds extends the previous work presented. ^11^ Future work will focus on expanding protocol coverage, implementing federated learning for privacy-preserving threat intelligence sharing, and conducting longitudinal studies on educational effectiveness.

## Source data

The datasets used for model training are publicly available:

• CIC-IDS2017 Dataset: Canadian Institute for Cybersecurity. Available at: https://www.unb.ca/cic/datasets/ids-2017.html
• CIC-DDoS2019 Dataset: Canadian Institute for Cybersecurity. Available at: https://www.unb.ca/cic/datasets/ddos-2019.html

Workshop questionnaire data was collected anonymously in compliance with GDPR and is available in aggregated form within the project deliverables.

## Software availability

Source code is available under the following repositories:

1. Github: Enhanced DNN Anomaly Detectors: https://github.com/Daskalos-Apps/Anomaly-Detectors (MIT License)
  Archived source code at time of publication: https://doi.org/10.5281/zenodo.18400377 ^37^
2. Cybersecurity Chatbot Implementation: https://github.com/Daskalos-Apps/cybersecurity-chatbot (MIT License) -Archived source code at time of publication: https://doi.org/10.5281/zenodo.18393845 ^38^
3. Quantised Phi-4 Model: https://huggingface.co/daskalos-apps/phi4-cybersec-Q4_K_M - (License: MIT License)

## Ethical considerations

This research was conducted as part of the SentinelSphere project under the ResilMesh consortium (EU Horizon Europe Grant Agreement No. 101119681). The study design, participant recruitment, and data collection procedures were reviewed and approved through the consortium’s ethical oversight framework, which operates in accordance with the principles stated in the Declaration of Helsinki.

The research involving human participants was classified as minimal risk, involving only anonymous questionnaires assessing cybersecurity knowledge and platform usability. No sensitive personal data, clinical interventions, or vulnerable populations were involved. The study was conducted in compliance with the European Code of Conduct for Research Integrity and the General Data Protection Regulation (GDPR) 2016/679.

A Data Protection Officer (DPO) was appointed to oversee all aspects of data protection compliance ( dpo@daskalos-apps.com). All data handling practices were reviewed to ensure alignment with GDPR requirements and EU ethical standards for research integrity.

## Informed consent

Written informed consent was obtained from all participants prior to their involvement in the study. Before completing the questionnaires, participants were presented with a consent statement that outlined: (i) the purpose of the study to record their habits and opinions regarding cybersecurity and the use of artificial intelligence; (ii) that participation was voluntary and anonymous; (iii) that all data would be processed in compliance with GDPR; (iv) their right to withdraw at any time without consequence; and (v) that responses could not be withdrawn after submission due to anonymisation.

Participants confirmed their consent by proceeding with the questionnaire after reading and acknowledging the consent information. All participants were adults (18 years or older) and no minors were involved in the study. The anonymous nature of data collection meant that individual responses could not be linked to specific participants, ensuring privacy protection while enabling meaningful analysis of aggregated findings.

## Declaration of generative AI and AI-assisted technologies in the writing process

During the preparation of this manuscript, the authors used generative AI-based tools, specifically large language models such as ChatGPT (OpenAI), solely to assist with language editing and consistency checks in limited sections. All substantive decisions regarding the design of the review, screening, data extraction, analysis and interpretation were made by the authors. The authors carefully reviewed and edited all AI-assisted text and take full responsibility for the integrity and accuracy of the manuscript’s content.

## Data Availability

-Zenodo: SentinelSphere Workshop Questionnaire Data (Aggregated) https://doi.org/10.5281/zenodo.18400306. ^36^ The above workshop project contains the following underlying/Extended data:
•
SentinelSphere_Cybersecurity_Survey_Part1.csv (Aggregated pre-workshop questionnaire responses including technology competency, AI familiarity, cybersecurity awareness, security practices, and regulatory knowledge metrics) •
SentinelSphere_Cybersecurity_Survey_Part2.csv (Aggregated post-workshop questionnaire responses including presentation quality, platform engagement, chatbot performance, TLS comprehension, deployment interest, and satisfaction metrics) Data are available under the terms of the Creative Commons Attribution 4.0 International license (CC-BY 4.0). Note: Individual-level questionnaire responses cannot be shared due to the anonymous nature of data collection under GDPR compliance. Aggregated summary statistics are provided to enable verification of reported findings while protecting participant privacy.
