# Supplementary material for: SentinelSphere: An AI-driven cybersecurity platform integrating real-time threat detection with security awareness education
Source: Open Res Eur. 2026 Jun 13;6:58. Originally published 2026 Feb 25. [Version 2] doi: 10.12688/openreseurope.22957.2 (PMC13276484; doi:10.12688/openreseurope.22957.2)
Supplement: Peer review history

# Reviewer report: Reviewer response for version 2

**DOI:** 10.21956/openreseurope.26095.r75908

**Published:** 2026-06-19

**Authors:** Oluwaseun Oladeji Olaniyi

**Affiliations:** 1 University of the Cumberlands, Williamsburg, Kentucky, USA

This manuscript presents SentinelSphere, an AI-driven cybersecurity platform that integrates machine learning-based threat detection with LLM-powered cybersecurity education. The platform combines an enhanced deep neural network, a traffic-light threat visualization system, a Phi-4 cybersecurity chatbot, and a Rust-optimized processing pipeline. Validation was performed using benchmark intrusion detection datasets and workshops involving 79 stakeholders.

 The work addresses an important cybersecurity challenge by combining technical threat detection with user awareness training. The platform is well designed, practically relevant, and demonstrates promising performance and usability.

 Review Questions

1. Is the work clearly and accurately presented and does it cite the current literature?

Answer: Yes

 The manuscript is clearly written, logically organized, and supported by relevant and current literature. The authors effectively position their contribution within research on intrusion detection, cybersecurity education, LLMs, and cyber resilience.

1. Is the study design appropriate and does the work have academic merit?

Answer: Yes

 The study combines system development, benchmark evaluation, performance optimization, and stakeholder validation. The integrated approach has clear academic and practical value and addresses an important gap between technical security controls and human factors.

1. Are sufficient details of methods and analysis provided to allow replication by others?

Answer: Partly

 The manuscript provides substantial methodological information but lacks several details needed for full replication.

 Key issues:

 • Exact definitions of engineered features (e.g., Request Complexity Score, Response Pattern Ratio, Path Entropy) are not provided.

 • Dataset preprocessing, normalization, and train/validation/test split procedures are insufficiently described.

 • Survey instruments and workshop questionnaires are not included.

 Suggested improvements:

 • Provide mathematical definitions for engineered features.

 • Document preprocessing and data partitioning procedures.

 • Include questionnaires and evaluation materials as supplementary files.

1. If applicable, is the statistical analysis and its interpretation appropriate?

Answer: Partly

 Performance metrics are reported appropriately, but statistical rigor could be improved.

 Key issues:

 • No confidence intervals or variability estimates are reported.

 • No statistical significance testing is provided.

 • Workshop findings are largely descriptive.

 • The professional validation cohort (n=3) is too small for broad conclusions.

 Suggested improvements:

 • Report confidence intervals for key metrics.

 • Include repeated-run statistics where applicable.

 • Clarify that workshop findings are exploratory.

1. Are all the source data underlying the results available to ensure full reproducibility?

Answer: No

 Although the primary benchmark datasets are publicly available, the materials required for full reproducibility are not provided.

 Missing items include:

 • Feature extraction scripts.

 • Training and evaluation code.

 • Survey instruments.

 • Anonymized workshop data.

 • Experimental configuration files.

 Required action:

 The authors should provide code, supporting datasets, and evaluation materials through a public repository.

1. Are the conclusions drawn adequately supported by the results?

Answer: Yes

 The conclusions are generally supported by the reported results. Claims regarding detection performance, scalability, and user acceptance are justified by the presented evidence. The authors appropriately acknowledge the need for future longitudinal studies before claiming sustained behavioral change.

 Issues Requiring Attention

 To strengthen scientific transparency and reproducibility, the authors should:

1. Provide complete feature-engineering and model implementation details.
2. Describe dataset preparation and partitioning procedures in full.
3. Release survey instruments and evaluation materials.
4. Make code and experimental configurations publicly available.
5. Provide anonymized validation datasets where possible.

Overall Assessment

 This is a strong and timely contribution that successfully combines AI-based threat detection with cybersecurity awareness education. The manuscript is scientifically sound, well written, and demonstrates practical value. The primary weakness is limited reproducibility rather than flaws in the research itself. Addressing the transparency and replication issues would further strengthen the work.

 Recommendation: Approved with reservations

Is the study design appropriate and does the work have academic merit?

Yes

Is the work clearly and accurately presented and does it cite the current literature?

Yes

If applicable, is the statistical analysis and its interpretation appropriate?

Partly

Are all the source data underlying the results available to ensure full reproducibility?

No

Are the conclusions drawn adequately supported by the results?

Yes

Are sufficient details of methods and analysis provided to allow replication by others?

Partly

Reviewer Expertise:

Cybersecurity, Cyber Engineering, Information Governance, Data Privacy, Risk Management, Artificial Intelligence Applications in Security, Security Awareness and Human Factors, Information Systems, Data Analytics, Digital Governance, Cyber Resilience, Threat Detection and Incident Response, Enterprise Risk Management, Compliance and Regulatory Frameworks.

I confirm that I have read this submission and believe that I have an appropriate level of expertise to confirm that it is of an acceptable scientific standard, however I have significant reservations, as outlined above.

# Reviewer report: Reviewer response for version 2

**DOI:** 10.21956/openreseurope.26095.r75909

**Published:** 2026-06-19

**Authors:** Hassan Rehan

**Affiliations:** 1 Purdue University, West Lafayette, Indiana, USA

The paper presents SentinelSphere, a cybersecurity platform that integrates AI-driven threat detection with LLM-powered security awareness education, built as an extension to the EU Horizon Europe ResilMesh framework. The detection component uses an Enhanced Deep Neural Network with 31 features (21 standard network features plus 10 HTTP-specific indicators) trained on the CIC-IDS2017 and CIC-DDoS2019 datasets, reporting 94% F1 score and a 69.5% reduction in false positives over the ResilMesh baseline. The educational component uses Microsoft's Phi-4 (14B parameters) quantized via Q4_K_M / llama.cpp / GGUF to 2.5 GB, enabling CPU-only deployment on standard hardware. A Rust rewrite of the core detection logic delivers 5.6× speedup for steady-state workloads and up to 326× for large batch processing. The system was validated through two workshops in Greece: a small professional cohort (N=3, multi-sector) and an educational cohort (N=76, predominantly undergraduate pedagogy students). All source code, the quantized model, and aggregated workshop data are openly published.

 The v2 revision has appropriately addressed the major concerns raised by the previous reviewers, including clarification of participant cohorts (new Table 6), full specification of the Phi-4 variant and quantization method, technical explanation of the 326× Rust speedup, explicit treatment of class imbalance handling, and softening of behavioral-change claims to match what the workshop data can actually support. The work is genuinely useful, the integration of detection and education is novel in practice, and the open availability of code and model is exemplary. The reservations below are about strengthening the empirical case rather than questioning the core contribution.

**Methods and Replication**

 The Phi-4 specification, the Rust rewrite explanation, and the class imbalance treatment are all sufficient for replication after the v2 revisions. Two gaps remain. First, the Enhanced DNN is benchmarked only against the original ResilMesh anomaly detection baseline. The paper positions its results favourably against published numbers from Khan et al. (92% precision with attention mechanisms) and Ferrag et al. (typical 5-15% false positive reduction for DNN approaches), but no head-to-head comparison is run on the same data splits with the same evaluation protocol. For a paper that claims to exceed prior work, at least one external comparison on the same dataset would substantially strengthen the empirical case. The contractive autoencoder result referenced from Aktar and Nur (97.58% accuracy on CIC-DDoS2019) is one obvious candidate. Second, the training/test split for the Enhanced DNN is not described — was the model evaluated through stratified k-fold, a single hold-out split, or temporal splitting? Given the extreme class imbalance, this matters for interpreting the reported metrics.

**Statistical Analysis**

 The standard classification metrics (F1, precision, recall, accuracy, confusion matrices) are appropriately applied for the multi-class detection task, and the prior reviewer is correct that the 69.5% false positive reduction is large enough that statistical significance testing is not the central concern. Three issues warrant attention.

 First, the SQL injection detection row in Table 4 reports an improvement from 85.7% to 90.5%, but with only 21 samples in this class, this represents detecting 19 of 21 samples versus 18 of 21 samples — a difference of one sample. The "4.8% improvement" framing significantly overstates what can be inferred from this comparison. The authors should either report this as 18/21 vs 19/21 to convey the actual count, or add a caveat that the SQL injection class is too small to support meaningful percentage comparisons.

 Second, the educational workshop's pre/post design is anonymous and therefore unpaired (N=39 pre, N=36 post). The v2 revision correctly acknowledges this in the body and softens corresponding claims, but the abstract still reports "most participants achieving improved security concept comprehension post-training," which is a comparison-of-means inference that anonymous unpaired samples can only weakly support. Either the abstract language should be further softened to match the body, or the framing should shift to baseline gaps identified versus post-workshop satisfaction reported.

 Third, the 91.7% Traffic Light System comprehension result is reported as a headline finding throughout the paper, but it is essential to note that this was measured on a cohort of mostly 18-20 year old pedagogy students with very low baseline cybersecurity expertise. A traffic light metaphor is intuitive by design and the cohort is exactly the audience for whom comprehension would be expected to be high. The result is not invalid, but it does not support generalization to SOC analysts, technical decision-makers, or other professional audiences. The professional workshop (N=3) is too small to bridge this gap. Either the framing should be more careful or the limitation should be discussed more explicitly.

**Summary**

 The technical conclusions about detection performance, false positive reduction, Rust optimization, and enterprise-scale event throughput are well supported by the results presented. The conclusions about the human-factor component are appropriately calibrated in the body of the v2 revision — the dedicated "From Comprehension to Behavior Change" subsection is well written and honest about what was and was not measured. However, some residual mismatches remain between the conclusions section and the body. The conclusion states the platform "demonstrates strong potential to address both technical detection and human security awareness challenges," but the human security awareness side of that claim is currently supported by short-term satisfaction and comprehension data from a single non-representative cohort, not by evidence of awareness improvement. The body acknowledges this; the conclusion could be tightened to match.

 A few smaller items. The text on page 4 cites the "Verizon 2023 Data Breach Investigations Report" while reference 5 lists the 2025 edition — the year should be reconciled. The plain language summary states the Rust rewrite is "more than five times faster" while the abstract and contributions feature the 326× figure for large batches; readers moving between sections may find this inconsistent. A short note in the plain language summary that the 5.6× figure refers to typical workloads while 326× applies at very large batch sizes would resolve this.

**Strengths**

 The integration of detection and education is genuinely valuable and rare in the literature. The Rust rewrite is a real engineering contribution and the technical explanation added in v2 (no GIL, contiguous memory layout, deterministic deallocation) is well grounded. The open availability of code, model, and data on GitHub, HuggingFace, and Zenodo is exemplary for this kind of work and sets a useful reproducibility standard for the field. The GDPR-compliant ethics handling is appropriate. The honest discussion of the gap between immediate comprehension and durable behavioral change, together with a concrete plan for longitudinal follow-up, is exactly the right way to handle this limitation.

**Items that should be addressed**

1. Add at least one external benchmark comparison on CIC-IDS2017 or CIC-DDoS2019 to substantiate the claim of exceeding prior work, or soften the comparative framing in the Related Work and Comparison with Related Work sections.
2. Describe the train/test split protocol used for the Enhanced DNN evaluation, including whether the reported metrics are from a single split or cross-validation.
3. Add a caveat to the SQL injection detection result in Table 4 indicating that the 21-sample class size limits the inferential weight of percentage-point comparisons; consider reporting raw counts.
4. Reconcile the Verizon 2023 vs 2025 reference between the text and the reference list.
5. Bring the abstract claim about post-training comprehension into closer alignment with the unpaired anonymous nature of the survey data, as already discussed in the body.
6. Add a sentence in the conclusion or limitations clarifying that the 91.7% TLS comprehension figure is from a low-baseline pedagogy cohort and may not generalize to professional audiences without further validation.
7. Reconcile the "more than five times faster" framing in the plain language summary with the 326× headline figure elsewhere by noting which figure applies to which workload.

**Overall**

 This is a substantial and useful piece of applied cybersecurity work with a real implementation, open artefacts, and honest reporting of limitations. The v2 revision has addressed the previous reviewers' main concerns thoroughly. The reservations above are about strengthening the empirical case and tightening some framing rather than questioning the substance of the work, and most are addressable in a focused minor revision. The paper deserves to be approved with these reservations addressed.

Is the study design appropriate and does the work have academic merit?

Yes

Is the work clearly and accurately presented and does it cite the current literature?

Yes

If applicable, is the statistical analysis and its interpretation appropriate?

Partly

Are all the source data underlying the results available to ensure full reproducibility?

Yes

Are the conclusions drawn adequately supported by the results?

Yes

Are sufficient details of methods and analysis provided to allow replication by others?

Partly

Reviewer Expertise:

Artificial Intelligence and Cybersecurity, with focus on deep learning for intrusion detection, federated learning, privacy-preserving analytics, and LLM applications in security

I confirm that I have read this submission and believe that I have an appropriate level of expertise to confirm that it is of an acceptable scientific standard, however I have significant reservations, as outlined above.

# Reviewer report: Reviewer response for version 2

**DOI:** 10.21956/openreseurope.26095.r75844

**Published:** 2026-06-17

**Authors:** Hewa Majeed Zangana

**Affiliations:** 1 Duhok Technical College, Duhok Polytechnic University, Duhok, Iraq

The authors have successfully addressed all of my previous concerns and reservations. Specifically, the addition of the detailed participant cohort table (Table 6) provides the necessary demographic clarity, the exact parameters and quantization framework for Microsoft's Phi-4 model have been clearly specified, all technical acronyms have been systematically defined, and the claims regarding long-term human-factor behavioral change have been appropriately softened to match the current scope of the workshop data. Furthermore, the technical explanation provided for the $326\times$ Rust-based speedup at scale is highly robust and scientifically sound.

 The manuscript is now exceptionally well-detailed, technically sound, and fully reproducible. I have no further comments to make, and I am pleased to recommend the paper for immediate acceptance and indexing in its current form.

Is the study design appropriate and does the work have academic merit?

Yes

Is the work clearly and accurately presented and does it cite the current literature?

Yes

If applicable, is the statistical analysis and its interpretation appropriate?

Yes

Are all the source data underlying the results available to ensure full reproducibility?

Yes

Are the conclusions drawn adequately supported by the results?

Yes

Are sufficient details of methods and analysis provided to allow replication by others?

Yes

Reviewer Expertise:

NA

I confirm that I have read this submission and believe that I have an appropriate level of expertise to confirm that it is of an acceptable scientific standard.

# Reviewer report: Reviewer response for version 2

**DOI:** 10.21956/openreseurope.26095.r75845

**Published:** 2026-06-15

**Authors:** Aryendra Dalal

**Affiliations:** 1 Middle Georgia State University, Macon, Georgia, USA

All previous comments have been addressed as required, no further comments

Is the study design appropriate and does the work have academic merit?

Yes

Is the work clearly and accurately presented and does it cite the current literature?

Yes

If applicable, is the statistical analysis and its interpretation appropriate?

Yes

Are all the source data underlying the results available to ensure full reproducibility?

Yes

Are the conclusions drawn adequately supported by the results?

Partly

Are sufficient details of methods and analysis provided to allow replication by others?

Partly

Reviewer Expertise:

Cybersecurity

I confirm that I have read this submission and believe that I have an appropriate level of expertise to confirm that it is of an acceptable scientific standard.

# Reviewer report: Reviewer response for version 1

**DOI:** 10.21956/openreseurope.24826.r71580

**Published:** 2026-03-27

**Authors:** Aryendra Dalal

**Affiliations:** 1 Middle Georgia State University, Macon, Georgia, USA

OVERALL RECOMMENDATION

 This paper tackles an important problem—combining technical threat detection with human security awareness—and does so with a practical, well-executed system. The work is fundamentally sound and makes genuine contributions. However, there are some gaps in how the evaluation is presented and a few technical details that need clarification before I can fully endorse it for indexing.

**Recommendation:** Approved with reservations (Minor revisions required)

 WHAT WORKS WELL

**The core idea is smart.** Most cybersecurity tools treat detection and education as completely separate problems. Your approach of using every security event as both a threat to neutralize and a teaching moment is genuinely novel and makes practical sense.

**The technical execution is solid.** The Rust rewrite delivering 5-6x speedup while maintaining 100% accuracy equivalence shows careful engineering. The 69.5% false positive reduction is meaningful—that's the difference between a tool analysts might actually use versus one they'll ignore due to alert fatigue.

**The validation effort is substantial.** You didn't just build a system and call it done. You actually put it in front of 76 real people across different sectors and measured their responses. The 86.1% satisfaction rate and 91.7% comprehension of your Traffic Light System are strong indicators the design works.

**Reproducibility is excellent.** Providing code on GitHub, the quantized model on HuggingFace, and aggregated data on Zenodo sets a good standard. Other researchers can actually build on this work.

 ISSUES THAT MUST BE FIXED

 1. **Workshop Participant Details Are Too Vague**

 You mention "76 stakeholders" and "54 participants" at different points, with pre-workshop N=39 and post-workshop N=36. This is confusing.

**What's actually unclear:**

- How many people attended the professional workshop versus the educational workshop?
- What was the baseline cybersecurity expertise of each group?
- Why did participant numbers drop from 39 to 36 post-workshop?

**Why this matters:** Saying "91.7% found the Traffic Light System clear" means something very different if you're talking about security professionals versus college students studying pedagogy. The reader can't properly interpret your validation results without knowing who these people actually were.

**Fix required:** Add a clear table showing:

- Workshop 1 (Professional): N=X, sectors represented, prior security experience
- Workshop 2 (Educational): N=76 (39 pre-survey, 36 post-survey), student vs. faculty breakdown, why some didn't complete both surveys

2. **The LLM Implementation Needs Clearer Specification**

 You say you used "Microsoft's Phi-4 model" with "Q4_K_M quantisation" but this is ambiguous.

**What's missing:**

- Which specific Phi-4 variant? (Phi-4-mini? Phi-4-base? Something else?)
- What was the exact model size before quantization (you say "28 GB to 2.5 GB" but Phi-4-mini is typically around 14GB)
- Which quantization framework did you use? (llama.cpp, GGUF, something else?)

**Why this matters:** LLM behavior varies significantly between versions. Someone trying to replicate your educational chatbot needs to know exactly which model to download.

**Fix required:** State explicitly in the text (not just the HuggingFace link): "We used Phi-4-mini-instruct (14B parameters, original size 28GB) quantized to Q4_K_M format using llama.cpp, resulting in a 2.5GB deployment model."

 3. **"Educational Retention" Is Mentioned But Not Measured**

 Your workshop shows people liked the chatbot and understood the traffic light system immediately after seeing it. But does this actually change their behavior over time?

 You acknowledge this limitation briefly but it deserves more discussion because it's central to your claimed contribution.

**Current state:** You measured immediate comprehension and satisfaction. You didn't measure whether people actually started using 2FA, stopped reusing passwords, or became better at spotting phishing emails weeks or months later.

**Why this matters:** The cybersecurity education literature you cite (Bada et al., Aldawood & Skinner) specifically points out that awareness campaigns often fail to change actual behavior. Your system might be better, but you haven't shown that yet.

**Fix required:** Add a paragraph in the Discussion or Limitations section explicitly stating:

- This study measured immediate comprehension and satisfaction
- Actual behavior change (e.g., adoption of 2FA, reduced password reuse) was not measured
- Longitudinal studies tracking participants over 3-6 months are needed to validate educational effectiveness
- This is a priority for future work

4. **Some Acronyms Are Not Defined at First Use**

 Examples I caught:

- "RNN" appears on page 5 without definition
- "CNN-LSTM" appears without spelling out what CNN and LSTM stand for
- "SOAR platforms" mentioned in Future Work without definition

**Why this matters:** Your paper bridges cybersecurity and education domains. Not all readers will know every machine learning acronym.

**Fix required:** Go through the entire manuscript and ensure every technical abbreviation is defined the first time it appears. This is a simple editing pass.

 QUESTIONS AND CLARIFICATIONS

**Q1: The 326x speedup for 100,000 events—is that real?**

 Your Table 2 shows processing 100,000 events in 5.39 seconds (Rust) versus 1,756.9 seconds (Python). That's a 326x improvement which seems almost too good to be true.

 I'm not saying it's wrong—Rust absolutely can deliver these gains for I/O-heavy workloads. But it would be helpful to explain what specifically caused such a dramatic difference. Is this mostly eliminating Python's Global Interpreter Lock for parallel processing? Better memory layout? Something else?

 A one-sentence technical explanation would make this more credible.

**Q2: How did you handle the class imbalance in training?**

 You mention 168,186 benign samples versus only 21 SQL injection samples. That's an extreme imbalance. Did you:

- Use class weights during training?
- Apply SMOTE or other oversampling?
- Just accept the imbalance and focus on precision/recall for minority classes?

This matters for understanding how your model actually works.

 MINOR WRITING ISSUES

**The "Plain Language Summary" is excellent** —genuinely accessible while still being accurate. Keep that.

**Some sentences are unnecessarily complex.** Example from page 4: *"The architecture operates as a strategic enhancement to the ResilMesh security framework, integrating as an advanced threat analytics layer while preserving the existing architecture's integrity."*

 Simpler version: *"SentinelSphere extends the ResilMesh framework by adding advanced threat analytics without disrupting existing workflows."*

 Not a big deal, but simpler prose helps international readers.

 WHAT ABOUT THE STATISTICAL ANALYSIS?

 The statistics you present are appropriate for this type of work:

- Standard classification metrics (F1, precision, recall, accuracy)
- Confusion matrices properly constructed
- Performance benchmarks with clear methodology

No issues here. You're comparing against a well-defined baseline (the original ResilMesh detector) and the improvements are substantial enough that statistical significance testing isn't really necessary—a 69.5% false positive reduction is clearly meaningful.

 DATA AVAILABILITY

 You've done everything right here:

- Public datasets clearly referenced (CIC-IDS2017, CIC-DDoS2019)
- Code on GitHub with DOIs
- Model on HuggingFace
- Aggregated workshop data on Zenodo

The explanation of why you can't share individual survey responses (GDPR compliance, anonymous collection) is reasonable and well-stated.

 No changes needed.

 DO THE CONCLUSIONS FOLLOW FROM THE RESULTS?

 Mostly yes, with one caveat.

**Claims that are well-supported:**

- Enhanced DNN achieves better detection with fewer false positives ✓
- Rust implementation delivers major performance gains ✓
- System can scale to enterprise workloads ✓
- Users find the Traffic Light System intuitive ✓
- Workshop participants were satisfied with the platform ✓

**Claim that needs softening:**

- "The system addresses both technical and human-factor cybersecurity challenges"

You've shown it addresses the technical detection challenge effectively. For the human factor, you've shown people like the educational component and understand it immediately after seeing it. But you haven't shown it actually changes security behavior over time.

**Fix:** Change this to "The system demonstrates potential to address both technical detection and human security awareness challenges, though longitudinal studies are needed to validate sustained behavioral change."

 SUMMARY OF REQUIRED CHANGES

**Must fix for scientific soundness:**

1. **Clarify workshop demographics** - Table showing participant counts, expertise levels, and sector breakdowns for both workshops
2. **Specify LLM model exactly** - State precise model variant, original size, and quantization method in the text
3. **Acknowledge educational effectiveness limitation** - Add explicit discussion that you measured immediate satisfaction but not long-term behavior change
4. **Define all acronyms** - Edit pass to ensure RNN, CNN-LSTM, SOAR, etc. are defined at first use

**Should fix to strengthen the paper:**

1. Explain what causes the dramatic 326x speedup for large batches
2. Clarify how you handled class imbalance during training
3. Soften the claim about addressing "human-factor challenges" to reflect that behavior change wasn't measured

FINAL ASSESSMENT

 This is good work that makes genuine contributions to an important problem. The technical system is well-designed, the performance improvements are substantial, and the integration of education with detection is genuinely novel.

 The issues I've raised are fixable with revisions—none of them undermine the core findings. Once you address the participant demographics,

 LLM specification, and educational effectiveness limitations, this will be a solid contribution to the cybersecurity literature.

 The work deserves indexing after these revisions.

Is the study design appropriate and does the work have academic merit?

Yes

Is the work clearly and accurately presented and does it cite the current literature?

Yes

If applicable, is the statistical analysis and its interpretation appropriate?

Yes

Are all the source data underlying the results available to ensure full reproducibility?

Yes

Are the conclusions drawn adequately supported by the results?

Partly

Are sufficient details of methods and analysis provided to allow replication by others?

Partly

Reviewer Expertise:

Cybersecurity

I confirm that I have read this submission and believe that I have an appropriate level of expertise to confirm that it is of an acceptable scientific standard, however I have significant reservations, as outlined above.

# Reviewer report: Reviewer response for version 1

**DOI:** 10.21956/openreseurope.24826.r70373

**Published:** 2026-03-13

**Authors:** Hewa Majeed Zangana

**Affiliations:** 1 Duhok Technical College, Duhok Polytechnic University, Duhok, Iraq

The manuscript presents a timely integration of AI-driven threat detection with real-time security education. By utilizing an "Enhanced DNN" for network traffic analysis and an LLM for generating contextual educational content, the authors address both technical defense and the "human factor" vulnerability. The transition from Python to Rust for performance optimization is a practical contribution that enhances the paper's technical depth.

**Detailed Critical Assessment**

**1. Presentation and Literature Review** The work is clearly presented. The authors successfully bridge two often-disparate fields: machine learning for intrusion detection and pedagogical strategies for cybersecurity awareness. The citation of the 2024 NIST Cybersecurity Framework 2.0 demonstrates that the literature review is current.

- *Requirement for Soundness:* Ensure all acronyms (e.g., RNN, CNN-LSTM, LLM) are defined at first mention to maintain accessibility for interdisciplinary readers.

**2. Study Design and Academic Merit** The hybrid approach—treating a security alert as a "teachable moment"—is conceptually strong. The study design appropriately benchmarks the proposed DNN against the ResilMesh baseline.

- *Constructive Criticism:* While the study includes a "multi-sector stakeholder validation," the sample size and specific demographics of the workshop participants are slightly opaque. To strengthen the academic merit, the authors should explicitly state the number of participants and their prior expertise levels to better contextualize the "90% positive feedback."

**3. Methodology and Replication** The methodology is detailed, particularly regarding feature engineering (HTTP-specific indicators). Providing the source code via GitHub and the LLM via Hugging Face is a gold standard for reproducibility.

- *Requirement for Soundness:* The authors should specify the exact version/checkpoint of the LLM used (e.g., Llama-3-8B-Instruct) within the text, as model behavior can vary significantly across versions.

**4. Statistical Analysis and Interpretation** The metrics (F1, Precision, Recall) are standard and correctly applied. The 69.5% reduction in false positives is a significant finding that is well-supported by the dual-stage filtering logic described.

**5. Data Availability** The use of Zenodo for workshop data and the reliance on public datasets (CIC-IDS2017) fulfills open-science requirements. No further action is needed here.

**6. Support for Conclusions and Limitations** The conclusions follow logically from the results. The authors honestly address the "LLM hallucination" risk and the need for quantization.

- *Constructive Criticism:* The paper would benefit from a more rigorous discussion on the "educational retention" of users. While the platform *provides* education, the study does not yet prove long-term behavior change. Adding a "Future Work" note regarding longitudinal studies on user behavior after interacting with SentinelSphere would round out the discussion.

**Summary of Required Revisions for Scientific Soundness:**

1. **Quantify Stakeholders:** Clearly define the N-size and expertise of the workshop participants.
2. **Model Versioning:** Explicitly name the base LLM model and version used in the final implementation.

**Clarity:** Ensure all technical abbreviations are defined in the Introduction or Background sections.

Is the study design appropriate and does the work have academic merit?

Yes

Is the work clearly and accurately presented and does it cite the current literature?

Yes

If applicable, is the statistical analysis and its interpretation appropriate?

Yes

Are all the source data underlying the results available to ensure full reproducibility?

Yes

Are the conclusions drawn adequately supported by the results?

Yes

Are sufficient details of methods and analysis provided to allow replication by others?

Yes

Reviewer Expertise:

NA

I confirm that I have read this submission and believe that I have an appropriate level of expertise to confirm that it is of an acceptable scientific standard, however I have significant reservations, as outlined above.
